# Bloc atrio-ventriculaire transitoire au cours d'un goitre multinodulaire: à propos d'un cas

**DOI:** 10.11604/pamj.2014.19.53.5113

**Published:** 2014-09-23

**Authors:** Georges Rosario Christian Millogo, Lassina Konaté, André Samandoulougou, Yibar Kambiré, Nobila Valentin Yaméogo, Koudougou Jonas Kologo, Caleb Tindano, Jean Yves Toguyeni, Patrice Zabsonré

**Affiliations:** 1 Service de Cardiologie, CHU Yalgado, Ouédraogo, Burkina Faso

**Keywords:** Atrio-ventriculaire, goitre multinodulaire, hyperthyroïdique, atrioventricular, multinodular goiter, hyperthyroidism

## Abstract

Les troubles de conduction auriculo-ventriculaire sont relativement fréquents en Afrique grâce à l’élévation de l'expérience de vie dans nos sociétés en pleine transition épidémiologique. Ces troubles sont le plus souvent dus à une dégénérescence du tissu de conduction. La prise en charge des troubles conductifs (BAV3) consiste en l'implantation d'un stimulateur cardiaque définitif. Mais cette thérapeutique doit être précédée par une enquête étiologique pour détecter les autres causes de BAV transitoire. Nous rapportons l'observation d'une patiente de 75 ans, admise pour bloc auriculo-ventriculaire 2/1 et un goitre multi-nodulaire hyperthyroïdique; le traitement avec des antithyroïdiens de synthèse a vu la disparition du trouble conductif. Les BAV au cours de l'hyperthyroïdie sont rares, mais bien décrits. L’évolution est favorable sous traitement par antithyroïdiens de synthèse. Il ne faudrait donc pas implanter des pacemakers définitifs en première intention.

## Introduction

Le goitre multi nodulaire est la maladie thyroïdienne la plus fréquente dans le monde avec plus de 300 000 personnes affectées [1, 2]. Il est le plus souvent lié à un déficit en iode et est généralement euthyroidien. Cependant, certains goitres multi nodulaires peuvent devenir hyperthyroïdiens avec hyperfonctionnement autonome de certains nodules (9% environ) [3] responsable parfois d′une cardiothyréose. Les complications cardiaques sont de 3 ordres: l′insuffisance cardiaque, les anomalies du rythme ou de la conduction, les cardiopathies ischémiques. Si les anomalies du rythme sont fréquentes, celles de la conduction sont exceptionnelles. D'où l'intérêt de cette observation.

## Patient et observation

**Figure 1:**
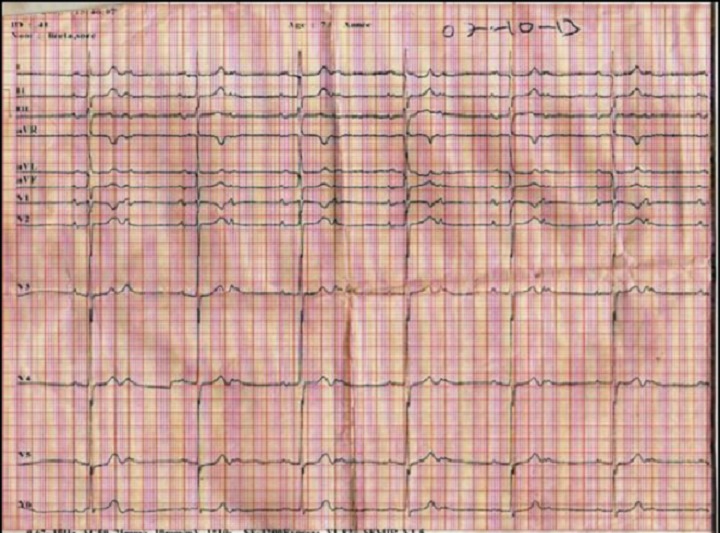
ECG douze dérivations réalisé le 07 Octobre 2013 mettant en évidence un BAV2/1 et BAV1

Mme SL, âgée de 75 ans était hospitalisée dans une Clinique de la place pour dyspnée d′effort et de décubitus mais sans syncope ni lipothymies. L′examen clinique a retrouvé des signes d′insuffisance cardiaque globale avec une bradycardie à 40/min, un souffle systolique éjectionnel en para sternal gauche, une pression artérielle à 190/70 mm Hg, un goitre d′environ 6 cm de grand diamètre, polylobé (que la patiente porte depuis plus de 40 ans). Un électrocardiogramme réalisé au lit du malade a montré un rythme atrial d′origine sinusal régulier à 75/min (intervalle P-P = 800 ms), un bloc atrio- ventriculaire de 2ème degré en 2/1 à QRS fins (Figure 1). L’échographie Doppler cardiaque montrait un ventricule gauche non dilaté (diamètre télé diastolique à 48 mm et diamètre télé systolique à 27 mmHg), un débit cardiaque estimé à 8 l/min, une FE VG (fraction d′éjection du ventricule gauche) de 75%. Elle a également noté une dilatation atriale gauche (surface = 24 cm^2^), des pressions de remplissage gauche élevées (E/E′ = 17), une bonne fonction systolique du ventricule droit avec une hypertension artérielle pulmonaire (PAPS estimées à 53 mm Hg).

Les analyses biologiques montraient un ionogramme sanguin normal, C Réactive Protéine = 4 mg/l, la Troponine I = 0,01, la créatininémie à 73,3 Umol/L. Le dosage des hormones thyroïdiennes montrait une TSH US < 0,05 mUI/L, une T3 libre = 10,4 ng/L et une T4 libre = 16,5 ng/L. Les autres examens thyroïdiens (échographie, scintigraphie, recherche des anticorps) n′ont pas été réalisés. Le diagnostic de goitre multi nodulaire avec hyperthyroïdie compliquée d′insuffisance cardiaque et bloc atrio-ventriculaire (BAV) de deuxième degré 2/1 probablement nodal fut posé. La patiente fut traitée avec du furosémide, du captopril et du néomercazole. L′évolution immédiate fut bonne avec une régression complète des signes d′insuffisance cardiaque, mais avec la persistance du BAV. L′hospitalisation a duré une semaine et l′évolution ultérieure était marquée par la normalisation progressive des hormones thyroïdiennes: TSH US < 0,05 en septembre et novembre 2013, puis 0,21 mUI/L en mars 2014.

**Figure 2:**
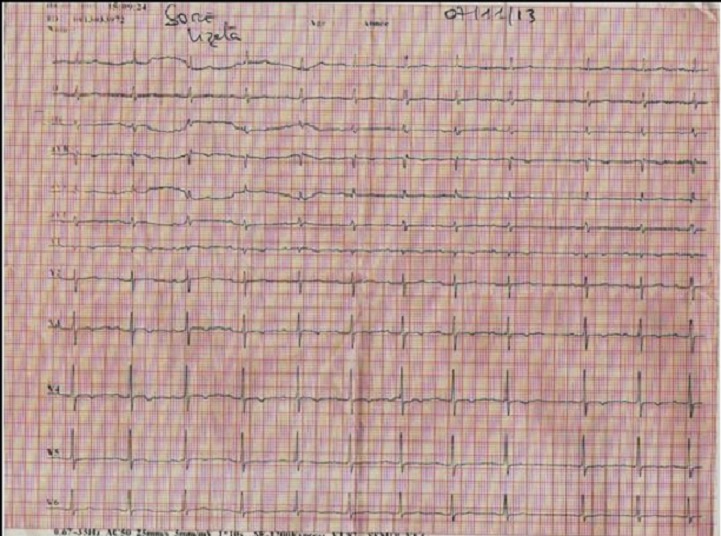
ECG douze dérivations réalisé le 07 Novembre 2013 mettant en évidence la disparition du BAV2/1, avec la persistance du BAV1 (PR= 22 ms)

**Figure 3:**
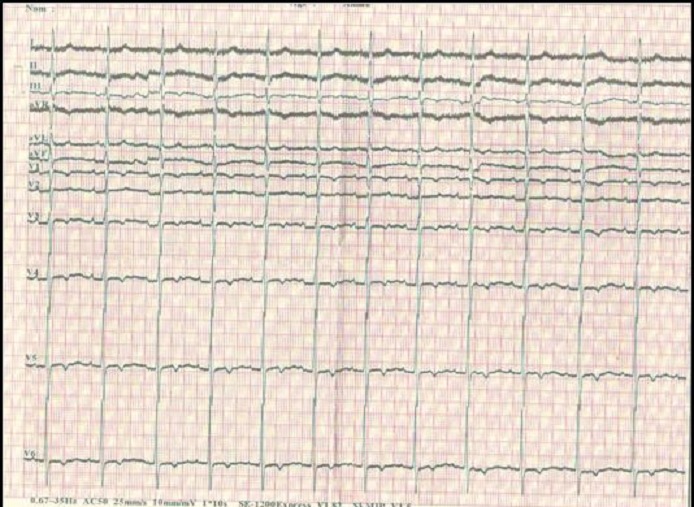
ECG douze dérivations réalisé le 10 Mars 2014 confirmant la disparition du BAV 1 (PR= 18 ms)

La thyroxinémie à 11,3 ng/l en septembre, normalisation à partir d′octobre 2013 (4,92 ng/ml et 6,92 ng/l en novembre). Sur le plan électrique, on a noté une conduction en 1/1 avec quelques passages en BAV 2 Mobitz 2 (en octobre 2013) puis une disparition complète du BAV sur 2 ECG successifs (novembre 2013 et mars 2014, voir la Figure 2 et la Figure 3). Tous les traitements à visée cardiaque furent arrêtés avec succès. Il s′agissait donc d′un BAV très probablement lié à l′hyperthyroïdie et complètement régressif après la correction de cette dernière. L′insuffisance cardiaque était liée à la fois à l′hyperthyroïdie et au BAV.

## Discussion

Les BAV par hyperthyroïdie sont très rares et de mécanismes peu connus. Un cas similaire au nôtre fut publié en 1987 par Archambeaux- Mouveraux [4]. Il s′agissait d′un patient avec hyperthyroïdie sévère qui présentait une alternance BAV 2 et BAV 3 sur une période de 6 ans. L′hyperthyroïdie a fini par être diagnostiquée et les anomalies conductives ont complètement disparu après retour en euthyroidie. Touloune F [5] a lui aussi rapporté le cas d′un sujet jeune atteint de la maladie de Basedow, qui avait à l′ECG un aspect BAV 1 et BAV 2 Mobitz 1. Ces anomalies conductives disparaissaient également après retour en euthyroidie. Trois autres études menées ont décrit les mêmes aspects.

Togaloglu C [6] en Turquie a récemment décrit 2 cas d′hyperthyroïdie avec BAV complet qui a disparu définitivement après guérison de la maladie thyroïdienne. Atri et al [7] en Inde ont aussi décrit un cas de BAV de 2ème degré dû à une hyperthyroïdie totalement régressive sous antithyroidien de synthèse. Déjà en 1980, Miller RH [8] avait compilé 35 cas de BAV par hyperthyroïdie qui étaient tous réversibles sous antithyroïdiens de synthèse. Ces cas ont été explorés par des examens électrophysiologiques endocavitaires et se sont avérés tous d′origine nodale. Il faut dire que la plupart des cas décrits étaient des maladies de Basedow. Ce qui avait fait évoquer par certaines équipes l′hypothèse d′une étiologie immunologique (myocardite auto-immune concomitante de la maladie de Basedow) [4]. Mais cette hypothèse n′a pas été confirmée, car aucun argument de myocardite n′a été retrouvé. En réalité, l′hyperthyroïdie est plutôt responsable de troubles du rythme supraventriculaires (2 à 20% selon les séries) surtout, ou ventriculaires. Un autre mécanisme a été évoqué sans être confirmé: l′hyperstimulation du nœud atrio- ventriculaire serait responsable d′un mécanisme réflexe d′hypervagotonie [5]. En réalité, les mécanismes des anomalies conductives au cours des hyperthyroïdies restent mal connus.

## Conclusion

Les BAV au cours de l'hyperthyroïdie sont rares, mais bien décrits. Il ne faudrait donc pas toujours se précipiter pour implanter des pacemakers définitifs. En dehors d'un contexte particulier, il faudrait d'abord penser l'hyperthyroïdie, surtout en présence de complexes QRS fins à l'ECG.
